# Covid-19 skepticism and public health norms during refugee assistance: does skepticism always lead to poor safety protocol adherence?

**DOI:** 10.1186/s12889-024-18232-3

**Published:** 2024-03-22

**Authors:** Stephanie J. Nawyn, Ezgi Karaoğlu, Natalie Qaji, Natalynn Qaji

**Affiliations:** Michigan State University, East Lansing, USA

**Keywords:** Refugees, COVID-19, COVID skepticism

## Abstract

**Background:**

Skepticism about COVID-19’s existence or severity has spread as fast as the disease itself, and in some populations has been shown to undermine protective public health behaviors that can mitigate infection. For populations that are especially vulnerable to COVID spread and severity, such as refugees, COVID skepticism is particularly problematic.

**Methods:**

We examine data collected from observations of humanitarian services provided to refugees in Lebanon, Türkiye, and Jordan to determine if skepticism is related to adherence to specific health-protective protocols (masking, social distancing, and hand sanitizing), and whether the effects of COVID skepticism are mediated by particular populations of refugees or the country in which those refugees receive assistance.

**Results:**

We found that community skepticism (the frequency of COVID skepticism expressed by others within a service location) is associated with lower adherence to certain protocols and not others. We also found that with certain protocols, the country in which refugees receive services mediates the relationship between community skepticism and protocol adherence, but for other protocols the relationship between skepticism and adherence is independent of either country in which refugees reside or the refugee population being served.

**Conclusions:**

The existence of skepticism about COVID-19 does not always lead to an unwillingness to take protective measures to avoid infection. The mechanisms underlying the relationship between skepticism and adherence to health-protective protocols vary based on the type of protocol in question. In order to increase protocol adherence, the specific variables predicting adherence to different protocols need to be assessed in order to increase adherence and improve public health during humanitarian services.

## Background

### Introduction

As knowledge of COVID-19 disease itself has expanded, attention has increasingly focused on how people’s perceptions of COVID-19 are affecting health-related behaviors and the spread of the disease. A large amount of misleading and false information about COVID-19, constituting what the World Health Organization calls an “infodemic” [1], increases the public’s uncertainty about how to respond to the disease and threatens to undermine sound public health policies. Research has connected people’s skepticism about COVID’s existence or severity to lower frequency of protective public health behaviors such as masking, social distancing, hand washing and sanitizing, and vaccination. This is especially problematic for refugees, who have higher vulnerability to COVID spread and severity [2].

In this paper, we examine data collected from observations of humanitarian services provided to refugees in Lebanon, Türkiye, and Jordan to measure the presence of COVID skepticism in service locations, to determine if skepticism is related to adherence to specific health-protective protocols (masking, social distancing, and hand sanitizing), and to test whether particular populations of refugees are more vulnerable than others to COVID skepticism and less likely to follow health protocols because of COVID skepticism.

### COVID skepticism and health behaviors

Skepticism, conspiracy beliefs, and misinformation about disease and illness are common during public health crises [3]. We use the term “COVID skepticism” to refer to any belief that COVID-19 either is not a real disease, that it is not as serious as most experts or authorities claim it to be, and/or that it cannot be successfully treated by the prevailing recommended treatments (including any number of COVID vaccines now available). It could include beliefs in conspiracies, belief in misinformation, or doubt in the veracity of accurate information because of mistrust in the source of that information (such as mistrust in government officials). COVID skepticism tends to correlate with more conservative or right-wing political ideology across a large number of countries [4, 5] and is associated with a lower perception of risk of COVID-19 [6].

COVID skepticism is also associated with less adherence to public health protocols intended to reduce disease spread [7–9]. The major concern about COVID skepticism is that it might lead to people being less likely to engage in behaviors that will decrease the spread of the disease. Many studies have found a negative relationship between COVID skepticism and adherence to infection risk mitigation protocols [6, 7, 10–12]. A number of studies have found that believing that COVID is a hoax or similar conspiracy beliefs are negatively associated with practicing infection risk mitigation [7, 9, 11, 13]. Belief in misinformation about COVID has also been found to be negatively associated with public health practices such as social distancing [14–16], and positively associate with engaging in risky social activities [14, 17], although Enders et al. [15] found that conspiracy beliefs had a more detrimental effect on risk mitigation behaviors than misinformation.

### Covid-19 skepticism and mistrust in government

A common finding across studies of COVID-19 skepticism is its relationship to mistrust in government and other authorities. Mistrust in government is positively associated with COVID-19 skeptic beliefs in India [18], Germany [19] South Korea [20], and England [21]. But there are differences in this relationship based on the type of government. Van Mulukom et al. [7] found that belief in COVID-19 conspiracies was positively associated with trust in government in the case of populist conservative governments such as Brazil, the UK, and the US, but negatively associated with trust in more liberal governments.

### Covid-19 skepticism and threat perception

Scholars also argued that expressing COVID-19 skepticism might be a mechanism for coping with uncertainty and threat [6, 22], which is a particular concern for refugee communities who experience high levels of uncertainty and overlapping economic, social, and physical risks. A sense of community may mediate the relation between COVID-19 skepticism, authority mistrust, and perceptions of uncertainty and risk. Where there is a strong sense of community solidarity, mistrust in government does not appear to predict low adherence to safety protocols or preventive measures. For instance, pro-democracy protests in Hong Kong might be mediating the lack of trust in the government, as residents report a strong sense of community solidarity and mobilization among organizations that led to increased compliance with the safety measures among the public [23]. In sum, mistrust in the government might be partially mitigated or entirely irrevelant to public health protocol adherence if group solidarity is strong and group norms support protocol adherence. If this affect works across groups, we might expect a similar higher compliance among Palestinian refugees, who because of their socio-political experiences tend to have high levels of group solidarity and distrust of outsiders [24].

### Studies on Jordan, Türkiye, and Lebanon

Relatively few COVID skepticism studies have examined the Middle East, particular ones measuring the association between skepticism and health behaviors. In Jordan, belief in COVID-19 conspiracy theories increased from 47.9% to 58.5% between April 2020 to December 2020, and researchers found a positive association between those conspiracy beliefs and vaccine hesitancy [22, 25]. Conversely, a study in Türkiye found no association between COVID-19 conspiracy beliefs and preventive measures [10]. In Lebanon, trust in information from the government was associated with lower beliefs in COVID-19 myths and false information [26]. However, the Lebanese people’s trust in their government has weakened with the impact of the pandemic in addition to the country’s political and economic fragility and the struggling healthcare system [27], which might result in a rise in COVID-19 conspiracy beliefs.

**Fig. 1:**
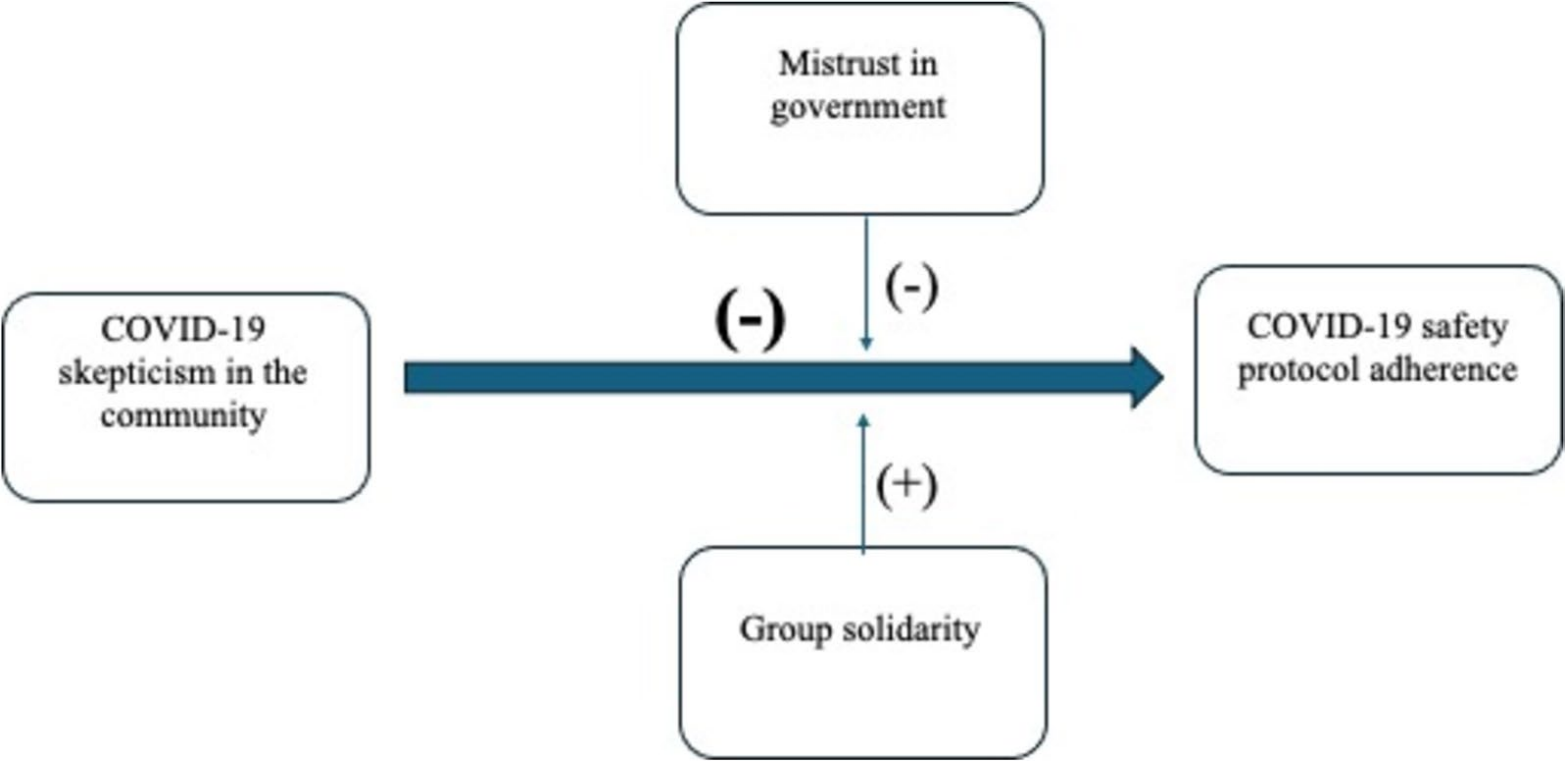
COVID community skepticism on COVID-19 safety protocol adherence

Based on the findings of past research, we conceptualize the relationship between community-level COVID skepticism and COVID safety protocol adherence as being negative and moderated by group solidarity and distrust in government. Distrust in government will negatively affect protocol adherence, while group solidarity will positively affect protocol adherence. Our conceptual model is described in Fig. 1.

While we do not have direct measures of distrust in government and group solidarity, based on past research and reports we can use services located in Beirut as a proxy for high government distrust (given the demands in Beirut for government officials to step down after the massive explosion in the Beirut port in August 2020), and services for Palestinians vs. Syrians as a proxy for group solidarity (given the literature demonstrating higher group solidarity among Palestinians).

Our study is novel in that it uses observational data collected during service provisions in humanitarian settings, thus does not rely on self-reports of public health behaviors. Through observing collective behaviors, including verbalizing COVID skepticism, we test the relationship between community skepticism and collective protocol adherence in a rare cross-country comparison.

## Methods

### Study design and data collection

This paper uses data collected through observations conducted during refugee humanitarian services in Türkiye, Jordan, and Lebanon. Four refugee assistance organizations partnered with US-based researchers; these were Amel Association and National Institution of Social Care and Vocational Training in Lebanon, Safa for Development in Türkiye, and Altkafal Charity Association in Jordan. The US-based researchers chose to invite these non-governmental organizations (NGOs) to collaborate based on their large caseloads of refugees, the diversity of services that they provided, and that medical services were included in those services. These NGOs are not necessarily representative of all humanitarian assistance NGOs in the region, although National Institution of Social Care and Vocational Training and Amel Association are the largest humanitarian assistance NGOs in Lebanon. Representatives from these organizations were full collaborators on the project, contributing to the research design and implementation, supervising the data collectors who conducted observations, and co-authored reports and peer-reviewed papers. The location of service centers in Türkiye was in Konya (central Türkiye) and Reyhanli (southeastern Türkiye). The service centers in Jordan were three locations in the governate of Irbid (northeastern Jordan). The service centers in Lebanon were dispersed throughout the country, with four locations in Beirut.

We hired data collectors who were native Arabic speakers to unobtrusively observe services provided by partnering NGOs. Staff were informed beforehand about the nature of the research and gave oral consent for the services they led to be observed. No observations were made during private services (such as medical or psychosocial) that would require patient consent, but data collectors did observe the waiting areas of those services. The data collectors recorded key information such as the number of people present services (counting refugees, staff, and visitors separately), the nature of the services, the primary refugee populations being served, and how many times they observed either refugees, staff, or other visitors violating social distancing, not wearing a mask, and how many times they observed people using hand sanitizer before, during, or after services. We also had data on hand washing with soap and water, but we present the findings here only on hand sanitizer use since it was more commonly available compared to soap and water. Data collectors also recorded if they overheard comments from either staff or refugees that expressed skepticism about COVID and the nature of those comments.

Between August 9 and September 15, 2020, data collectors recorded 215 observations. Data collectors recorded what service they were observing, how many people were in the space counting refugees, staff, and visitors separately, how often they observed refugees, staff, or visitors violating mask wearing or social distancing protocols, and how often they observed refugees/staff/visitors washing or sanitizing their hands. They also were asked to record any comments they overheard that they thought indicated the speaker did not take COVID-19 seriously, reporting comments from refugees and staff separately. They were then asked to label the comments within one of the following five categories:

1. “COVID-19 is a hoax/is not real” (for example, “Do not go to the hospital to get tested because they will kill you and say that you died of Corona”)
2. “COVID-19 is being exaggerated/is not real” (for example, “it is a normal flu that does not affect young people”)
3. “There are more serious problems than COVID-19 (such as having enough food to eat, having a safe place to live, etc.)” (for example, “the mask has become more important than eating or drinking”, referring to the difficulties of acquiring food in Lebanon).
4. “COVID-19 is an excuse for governments or other people to treat refugees poorly” (for example, a data collector overheard a woman say that COVID tests were faked because the government was collecting additional money for every refugee that tested positive)
5. “Other (please specify”)

Data collectors also recorded the location, day, and time of their observation, and which groups of refugees were primarily served. The observation data were recorded in Arabic and entered into Qualtrics in real-time. Data collectors also included open-ended notes about their observations. All Arabic entries were later translated into English and verified by bilingual speakers for accuracy.

While there were differences across the NGOs service centers in terms of layout, crowdedness, and seating arrangements, the data collectors were able to make and record their observations unobtrusively. Service centers were generally busy with many people sitting in waiting rooms and coming in and out of rooms during services, and so any one person sitting in the corner of a room would not be very noticeable. Additionally, our data collectors were natives and residents of the area around the service center and were mostly Arabic (including in Türkiye), and so were not easily distinguishable from the NGO staff.

### Variables

Our key independent variable is “community skepticism”, operationalized as whether or not any COVID skepticism was expressed by a person (either refugee service recipient or staff). Because the number of observations during which a staff member expressed COVID skepticism were small and were almost always accompanied by a refugee also expressing skepticism, we combined those two measures into a single measure of any COVID skepticism expressed. For other independent variables we used the measure of the origins of the refugees who were predominantly served in a particular service (primarily Syrians, primarily Palestinians, or other). Observers could choose both “primarily Syrians” and “primarily Palestinians.” We also used variables measuring the location in which the services took place (Türkiye, Jordan, Lebanon outside of Beirut, and Beirut), examining Beirut separately because of the massive explosion that occurred in the Beirut port on August 4, which dramatically increased the distrust in the Lebanese government, especially those living in Beirut.

For the dependent variables we used the number of people in physical space during services and the number of people violating or adhering to protocols and calculated a proportion variable (dividing the total number of people by the number of violations or adherences, depending upon the protocol). Our dependent variables were a) the number of social distancing violations by refugees, b) the number of mask-wearing violations by refugees, and c) the number of times refugees used hand sanitizer, all proportionate to the number of refugees present. Measuring protocol violation/adherence in this way controlled for the number of people present who could possibly violate or adhere to the protocols. All three protocols were required at the service locations but not evenly enforced. Soap and water were widely available during services (75% and 77% respectively across all observations). However, service centers were not always able to provide masks, the expense of which was a factor in refugee non-adherence.

### Statistical analysis

We used STATA (v.16.1) to run the analysis. After running univariate and bivariate analyses to examine the distribution of variables, we constructed three Ordinary Least Squares (OLS) regression models to test the relationship of COVID skepticism to protocol adherence and the effects of geographic location (which we use as a proxy for mistrust in government) and refugee population (which we use as a proxy for group solidarity). With these relationships in mind, we test the following hypotheses:

- *Hypothesis 1*) Community skepticism is negatively associated with protocol adherence. Service locations where any skepticism was observed will have higher proportions of refugees’ violating social distancing and mask wearing protocols, and a lower proportion of refugees using hand sanitizer, compared to service locations where skepticism was not observed.
- *Hypothesis 2*) The geographic location of services is related to protocol adherence, and moderates the relationship between community skepticism and protocol adherence. Because mistrust in the government was high in Beirut during the period of data collection, services provided in Beirut will have a greater proportion of refugees violating social distancing and mask wearing protocols and a lower proportion of refugees using hand sanitizer compared to services provided in Lebanon outside of Beirut, in Türkiye, and in Jordan. Adding the effects of geographic location in the model diminish the effects of skepticism on protocol adherence.
- *Hypothesis 3*) The population of refugees being served is related to protocol adherence, and moderates the effects of community skepticism. Because Syrian refugees have lower solidarity than Palestinian refugees, services for Syrians will have lower protocol adherence while services for Palestinians will have higher protocol adherence. These effects exist when controlling for geographic location of service provision, and diminish the effects of skepticism on protocol adherence.

While the NGOs contributing to the study were not randomly selected and thus their services are not representative of all humanitarian services in the entire region, we attempted to randomly select services within the total services provided by each NGO. We use T-tests of statistical significance with a 95% confidence interval to assess statistical significance in the regression models.

## Results

Our findings were mixed, with some hypotheses supported for certain protocols but not others. We describe the results in detail below.

### Descriptive statistics

Table 1 displays the distribution of skepticism by who expressed it (refugees or staff), and expressions of any skepticism by country and service recipients (mostly Syrians or mostly Palestinians). Observers frequently overheard refugees express skepticism about COVID-19 during their observations of services (31.31% of all observed services) but only occasionally overheard staff express skepticism (6.54% of all observed services), almost always when refugees did the same. This should not be surprising given that staff would likely have more public health training than refugees, and would feel constrained from expressing skepticism that might undermine their employers’ health protocols. For the remainder of the analysis we combine these two variables into a single measure of any skepticism expressed (by either refugees or staff). Across countries, skepticism was more prevalent in Beirut and Jordan compared to Türkiye and Lebanon outside of Beirut. In Beirut, 32.53% of total observations involved individuals that expressed some form of skepticism. In Jordan, 43.94% of total involved individuals that expressed some form of skepticism. Furthermore, when considering whether the services provided were largely for Syrians or Palestinians, more skepticism was observed during services for Palestinians (41.86% of total observations).

**Table 1:** Descriptive statistics

		%	Total observations
Overheard refugees express skepticism		31.31	215
Overheard staff express skepticism		6.54	215
Any skepticism expressed:			
	Overall	33.18	215
	Türkiye	27.59	29
	Jordan	43.94	53
	Lebanon	9.43	133
	Beirut	32.53	49
	During services primarily for Syrians	27.74	137
	During services primarily for Palestinians	41.86	86

### Skepticism predicting protocol adherence

We next test our hypotheses with nested OLS regression models, using any skepticism, location of service provision, and primary refugee population served to predict the proportion of refugees who violated mask wearing protocols and social distancing protocols, and the proportion of refugees who followed hand hygiene protocols at some point during service provision. Table 2 describes these findings.

- Hypothesis 1) Skepticism is negatively associated with protocol adherence. Service locations where any skepticism was observed will have higher proportions of refugees’ violating social distancing and mask wearing protocols, and a lower proportion of refugees washing their hands, compared to service locations where skepticism was not observed.

**Table 2:** Association of skepticism and geographic location to protocol adherence

Unstandardized regression coefficients predicting protocol adherence									
	Refugees Violating Mask Wearing Protocol			Refugees Violating Social Distancing Protocol			Refugees Using Hand Sanitizer		
	Model 1	Model 2	Model 3	Model 1	Model 2	Model 3	Model 1	Model 2	Model 3
	Coeff	Coeff	Coeff	Coeff	Coeff	Coeff	Coeff	Coeff	Coeff
Any Skepticism	0.11*	0.05	0.05	0.52*	0.60*	0.68**	0.00	0.09	0.09
Beirut (ref)		––	––		––	––		––	––
Lebanon Outside of Beirut		0.07	0.07		1.14***	0.98**		0.33**	0.34**
Türkiye		-0.03	-0.01		0.21	-0.27		0.25*	0.30*
Jordan		-0.22**	-0.92		0.26	-0.23		0.16	0.26*
Primarily Syrians			-0.03			0.70**			-.20*
Primarily Palestinians			0.02			-0.67**			-0.07
Constant	0.31***	0.36	0.36***	0.66***	0.10	0.14	.40***	0.18	.29**

^*^ *p* < .05

^**^ *p* < .01

^***^ *p* < .001

We tested Hypothesis 1 in Model 1. For mask wearing and social distancing protocols, any expressions of skepticism were found to be negatively related to protocol adherence. In service settings where observers overheard any skepticism, the proportion of refugees violating mask wearing was almost 11% higher compared to service settings where no skepticism was observed. For social distancing, observations of skepticism were associated with a nearly 52% increase in social distancing violations. Skepticism was not significantly related to the proportion of refugees’ following hand hygiene protocols.

- Hypothesis 2: The geographic location of services is related to protocol adherence, and moderates the relationship between skepticism and protocol adherence. Services provided in Beirut will have a greater proportion of refugees violating social distancing and mask wearing protocols and a lower proportion of refugees washing their hands compared to services provided in Lebanon outside of Beirut, in Türkiye, and in Jordan. Adding the effects of geographic location in the model diminish the effects of skepticism on protocol adherence.

We tested Hypothesis 2 in Model 2, using Beirut as the referent category for geography. Services provided in Jordan had significantly fewer mask wearing violations than in Beirut, and services provided in Lebanon outside of Beirut had significantly more social distancing violations but more adherence to hand hygiene protocols compared to Beirut. Services in Türkiye also had a higher proportion of refugees washing their hands compared to Beirut. For mask wearing violations, the effect of skepticism diminished to non-significance, indicating a moderating effect of service location. For social distancing violations, skepticism was still positively associated with the proportion of refugees who violated social distancing protocols; in fact, the size of the relationship increased.

- Hypothesis 3) The population of refugees being served is related to protocol adherence, and moderates the effects of skepticism. Services for Syrians have lower protocol adherence. Services for Palestinians have higher protocol adherence. These effects exist when controlling for geographic location of service provision, and diminish the effects of skepticism on protocol adherence.

We tested Hypothesis 3 in Model 3. We found that during services that included primarily Syrians there were more social distancing violations compared to services that primarily included other refugee groups. Conversely, services primarily including Palestinians had fewer social distancing violations. This controlled for the effect of location of services. The addition of primary refugee group served in the model increased the strength of skepticism on refugees’ violating social distancing protocols. In model 3, in services where skepticism was observed there was a 68% higher proportion of social distancing violations compared to services where skepticism was not observed.

Services to primarily Syrians also had less hand washing as well, and after controlling for population served all geographic locations had significantly more hand washing than Beirut. Refugee population served had no significant effect on face mask violations.

## Discussion

This study tested the hypotheses that expressions of COVID skepticism during humanitarian services to refugees was associated with lower adherence to COVID safety protocols, and that the relationship between skepticism and adherence was moderated by the country in which services were provided (a proxy for government mistrust) and the refugee receiving services (a proxy for group solidarity). Our findings overall indicate that skepticism has different effects on different types of protocol adherence. We found expressions of skepticism within a service area to be positively associated to mask wearing and social distancing violations, but not related to hand hygiene protocol adherence. Further, the geographic location of services modified the effect of skepticism for mask wearing but not social distancing.

Services in Lebanon were associated with higher levels of skepticism compared to Türkiye and Jordan, with skepticism being especially high in Beirut. Suggesting that political instability in Beirut and other parts of Lebanon might be increasing skepticism. However, skepticism does not always appear to be the main driver of lower COVID protocol adherence. Services in Jordan had fewer mask wearing violations and services in Türkiye and the rest of Lebanon had more hand sanitizer use than in Beirut, and with geographic region included in the model there was not significant effect of skepticism. Additionally, services in Beirut had fewer social distancing violations than the rest of Lebanon. We also found that services primarily for Syrians included more social distancing violations and less hand sanitizing, while services primarily for Palestinians and fewer social distancing violations. This supports part of Hypothesis 3 that Syrians would have worse protocol adherence and Palestinians would have better adherence, suggesting that a stronger sense of group solidarity among Palestinians compared to Syrians would facilitate protocol adherence regardless of skepticism.

It is possible that social distancing has costs associated with its practice that are different from mask wearing and hand hygiene. Mask wearing and hand hygiene can be practiced while still maintaining physical closeness to people, signaling social closeness and trust with those people. Service providers told us that many refugees felt like keeping a 2 m distance from another person was akin to stigmatizing that person, and was socially unacceptable. Therefore, maintaining social distancing may have required a stronger belief that the costs of social distancing were worth avoiding COVID infection, making adherence to that protocol more susceptible to COVID skepticism.

Conversely, hand hygiene was likely something that was done collectively (as in the case of everyone being asked to use hand sanitizer before entering a clinic), and did not involve the stigma of avoiding others or the discomfort of wearing a mask. Washing or sanitizing one’s hands was a practice that predated COVID, and has other benefits beyond avoiding COVID infection. Therefore, adherence to hand hygiene might not have been as susceptible to COVID skepticism as either mask wearing or social distancing.

## Limitations

A limitation of this study is that we cannot connect individual skepticism to individual behavior, nor could we directly measure sense of community. A study design that allowed for measuring individual beliefs and connected those to actual health behaviors (and not self-reported behaviors, which are likely to be biased when involving rule violations or showing disregard for the health of others) would be challenging. Our findings suggest that smaller-scale investigations of these dynamics are valuable, and can incorporate cross-national and population comparisons as well.

Because of the large number of locations where observations were recorded, our research team decided not to have multiple observers collecting data in one location simultaneously. Thus, we were not able to triangulate observations or measure inter-observer reliability. We collected data from each service location multiple times using different data collectors, and we anticipate that this practice would distribute any observer bias across equally across the locations. However, we are unable to verify that assumption.

## Conclusion

The existence of skepticism about COVID-19 does not always lead to an unwillingness to take protective measures to avoid infection. Our findings indicate that the mechanisms underlying the relationship between skepticism and adherence to health-protective protocols vary based on the type of protocol in question. Social distancing is negatively associated with skepticism, and that relationship remains after controlling for location of services (with parts of Lebanon outside of Beirut having more social distancing violations than within Beirut) and the refugee groups being served (with services for Syrians having higher rates of social distancing violations while services for Palestinians have lower rates of social distancing violations). Comparatively, mask wearing and using hand sanitizer are not so robustly related to skepticism. This suggests that improving adherence to public health protocols is more complicated than simply correcting skeptical beliefs about an infectious disease. For certain mitigation protocols, eliminating skepticism does not address the primary reasons that people receiving humanitarian assistance do not consistently follow those protocols.

Our findings also suggest that group solidarity in a given service affects protocol adherence. Among tightly-knit groups such as Palestinian refugees, protocol adherence is higher (at least with regards to social distancing). They also indicate that in places where distrust in government is highest (such as we assume exists in Beirut), protocol adherence is not necessarily worse than in other geographic locations. Future research that more directly measures government mistrust and group solidarity and culture is needed to identify how cultural norms shape public health protocol adherence.

Our findings complicate the negative relationship between COVID skepticism and COVID safety protocol adherence found in most of the literature. Different safety protocols likely have different costs associated with their practice. They could also have different cultural meanings to certain populations, as Kemmelmeier and Jami [28] found with regards to mask wearing. This may be why we found differences in the effects of community skepticism on different safety protocols. Service providers will need to understand the underlying dynamics of resistance to different protocols in order to assess the best strategies for overcoming the resistance. And additional research is needed to unpack how skepticism is moderated by other group characteristics in order to identify the best strategies for increasing safety protocols that mitigate infection risks, for COVID as well as the next pandemic.

## Data Availability

The datasets generated and used during the study are not publicly available due to privacy concerns of the non-governmental organizations that took part in this study. However, the de-identified data can be made available by the corresponding author upon reasonable request.
